# Making Sense of Genetic Information: The Promising Evolution of Clinical Stratification and Precision Oncology Using Machine Learning

**DOI:** 10.3390/genes12050722

**Published:** 2021-05-12

**Authors:** Mahaly Baptiste, Sarah Shireen Moinuddeen, Courtney Lace Soliz, Hashimul Ehsan, Gen Kaneko

**Affiliations:** School of Arts & Sciences, University of Houston-Victoria, Victoria, TX 77901, USA; BaptisteM1@uhv.edu (M.B.); smoinudd@wellesley.edu (S.S.M.); SolizCL1@uhv.edu (C.L.S.); ehsanh@uhv.edu (H.E.)

**Keywords:** breast cancer, glioma, precision medicine, single nucleotide polymorphisms

## Abstract

Precision medicine is a medical approach to administer patients with a tailored dose of treatment by taking into consideration a person’s variability in genes, environment, and lifestyles. The accumulation of omics big sequence data led to the development of various genetic databases on which clinical stratification of high-risk populations may be conducted. In addition, because cancers are generally caused by tumor-specific mutations, large-scale systematic identification of single nucleotide polymorphisms (SNPs) in various tumors has propelled significant progress of tailored treatments of tumors (i.e., precision oncology). Machine learning (ML), a subfield of artificial intelligence in which computers learn through experience, has a great potential to be used in precision oncology chiefly to help physicians make diagnostic decisions based on tumor images. A promising venue of ML in precision oncology is the integration of all available data from images to multi-omics big data for the holistic care of patients and high-risk healthy subjects. In this review, we provide a focused overview of precision oncology and ML with attention to breast cancer and glioma as well as the Bayesian networks that have the flexibility and the ability to work with incomplete information. We also introduce some state-of-the-art attempts to use and incorporate ML and genetic information in precision oncology.

## 1. Introduction

The Human Genome Project decoded over 3 billion nucleotides between 1990 and 2003, providing meaningful information to biomedical researchers [1]. The term *precision medicine* was introduced to the biomedical field in 1999 as one of the ways to utilize the human genome, while its basic principles go back to the 1960s [2]. Precision medicine refers to a medical approach in which treatments are tailored to individual patients and/or unique subpopulations—because the genetic variation among people directly impacts their susceptibility to diseases, prognoses, and response to treatment methods. Precision medicine has a high potential to offer effective preventative and therapeutic interventions limiting significant side effects to the unique patient.

A key discovery that has propelled the significant progress of precision medicine is the single nucleotide polymorphism (SNP) genotyping [2]. SNPs and copy number variations (CNVs) are responsible for roughly 0.9% of variation between individuals [3], being the main source of genetic difference between people. The human genome project led to the identification of about four to five million SNPs in the human genome, and SNPs located in or close to the genes and the regulatory regions gained particular interest due to their greater connection to human diseases (i.e., as the predictor for the risk of developing diseases) [4]. Furthermore, SNPs reveal important underlying differences that cause inter-individual pharmacokinetic variability [3,5]. Because of these substantial advantages of SNPs, many international projects were organized to build useful SNP databases. Starting in 2002, the International HapMap Project gathered common haplotypes from continents around the world to determine how SNPs impact the risk of diseases [6]. A map of common SNP patterns is now available in the public domain, facilitating the use of the information for increasing the accuracy of diagnosis and specificity of treatments for many diseases like cancer, diabetes, and cardiovascular diseases. In 2010–2012, the 1000 Genomes Project successfully profiled 1092 individuals’ genomes from 14 populations, capturing up to 98% of accessible SNPs [7,8], thus paving way for tailored detection, prevention, and treatment of diseases.

The primary methodologies of precision medicine include (1) identifying genes related to a particular disease and drug response, (2) predicting the risk of the disease based on the genetic information of subjects, and (3) addressing the technological issues involved in the treatment based on the genetic and phenotypic information of patients [9,10]. This approach works particularly well for cancers, in which family health history and genetic alterations have great impacts on risk prediction, diagnosis, and treatment. One of the pioneering projects is the collaboration between Perlegen Sciences, Inc. and Women’s Health Initiative in 2005, which employed high-density whole-genome scans of SNPs to assess the potential correlation between genetic predispositions for coronary heart disease, stroke, and breast cancer within 161,808 women between the ages of 50 and 79 undergoing postmenopausal hormone therapy [11]. In the same year, a collaboration between Cancer Research UK, the University of Cambridge, Cancer Research Technology, and Perlegen Sciences, Inc. determined over 200 million genotypes to “understand the genetic basis of the disease in the area of prevention, early detection, and treatment”, adding to our previous understandings relating breast cancer and hormone receptors [11].

In addition to the genetic variation of patients, the types of mutations in tumor cells greatly affect the prognosis and drug response. Precision medicine stemming from genetic variations in tumor cells (called “somatic mutations” hereafter) is typically called precision oncology. Recent advances in computational biology, such as bioinformatics and machine learning (ML), have provided effective aids in categorizing somatic mutations and making appropriate mathematical predictions [12,13]. Like the HapMap database for heritable SNPs, several databases have been established for somatic mutations. The major databases include The Cancer Genome Atlas (TCGA) (https://www.cancer.gov/tcga, accessed on 10 May 2021), the International Cancer Genome Consortium (ICGC) Data Portal (https://dcc.icgc.org, accessed on 10 May 2021), and the Catalogue of Somatic Mutations in Cancer (COSMIC) (https://cancer.sanger.ac.uk/cosmic, accessed on 10 May 2021). With these techniques, precision oncology, due to its specificity and tailored approach, will potentially be more beneficial for patients compared to one-drug-fits-all treatment methodologies [14].

In this narrative review, we aim to provide an overview of up-to-date precision oncology, including novel enlistment of computational biology, artificial intelligence (AI), and ML to better create targeted therapies for patients diagnosed with cancer. In this review, AI refers to a broader concept, a computational device to perform functions that are usually associated with human intelligence, whereas ML is a subset of AI that that is characterized by learned patterns derived from experiences (i.e., without being explicitly programmed) [15,16,17]. Special attention will be given to breast cancer and glioma, for which novel ML approaches have been actively employed.

## 2. The Path That Precision Oncology Has Taken

### 2.1. Overview

The accumulation of genetic data has opened a door of opportunities for predicting the risk of cancer in individuals with heritable cancer-causing variations in their genome, which increases the chance to implement preventive methods [18]. While early diagnostic screening through cytological methods has been effective in identifying types, subtypes, and the tumor-node-metastasis (TNM) stages of tumor cells, effective utilization of heritable genetic variation has the potential to facilitate even earlier intervention along with other clinical benefits [19,20]. This method is also useful for targeting treatment based on the person’s genome, to potentially optimize drug selection and reduce adverse side effects [21]. In this section, we summarize major accomplishments in precision medicine and precision oncology without the use of ML approaches.

In precision medicine, heritable genetic variations of subjects are assessed to determine whether they have a high risk of developing cancer [22]. Among various genetic variations, SNPs have been relatively well associated with developing breast cancer, glioma, or leukemia along with other forms of cancer [22,23,24,25,26]. The risk of cancer caused by these SNPs is heritable, accounting for approximately 10% of all cancer cases [27,28]. For example, SNPs have been used in pediatric subjects to determine the risk of acute lymphoblastic leukemia (ALL) [29] as well as the risk for developing thromboembolism, a major issue in ALL treatment [30]. Some SNPs can be associated with several types of cancers—many forms of cancer are poly-ADP ribose polymerase (PARP)-dependent, and polymorphisms in this gene are associated with the risk of various cancers [31,32]. The *UGT1A1* *28 polymorphism is a dosage indicator for the use of irinotecan [33]. Identifying common and diverse sets of SNPs in subjects thus leads to various quantitative analyses to keep track of and identify risk and outcome.

**Figure 1:**
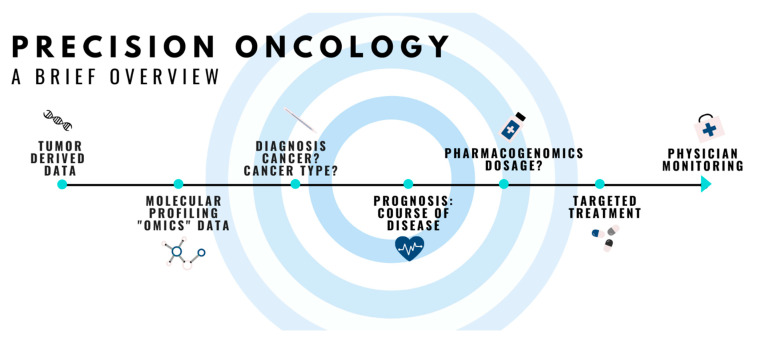
Overview of precision oncology.

Precision oncology investigates the somatic mutations in tumors to develop and/or apply targeted therapies according to the type of tumors [34,35] (Figure 1). Many tumors are dependent on the mitogen-activated protein kinase (MAPK) signaling, which is a conserved pathway responsible for organ development and tissue homeostasis in organisms, and variations in this pathway can be a target for precision oncology [36]. For low-grade gliomas, for example, the most common somatic mutation is a tandem duplication of 7q34 that results in the fusion of the *KIAA1549*-*BRAF* gene [24,37]. Several attempts have been made to establish targeted therapies for these tumors [38,39]. The somatic mutation in the *PARP1* is also known to affect the response to PARP inhibitors [5], helping to select a useful treatment strategy in various cancers. Other examples include epidermal growth factor receptor (EGFR) immunohistochemistry and *KRAS* proto-oncogene (*KRAS*) exon 2 mutation tests for determining the likelihood of treatment response to cetuximab or panitumumab treatment in metastatic colorectal cancer (CRC) [33]. Other molecular subtypes, such as SNPs in *KRAS* exon 3/4, *B-Raf* proto-oncogene, *NRAF*, *PIK3CA*, and *PTEN*, were also reported as potential new pharmacogenetic targets for the current and newly discovered anticancer drugs.

In addition to somatic mutations in tumors, gene and/or protein expression profiles can also be used to classify the type of tumors and to understand how patients respond to specific drugs [40]. The traditional microarray expression profiles that consisted of 92 genes identified two subgroups of tumors: those that were sensitive and resistant to docetaxel [41]. This technology has been upgraded to proteomics and protein microarray analysis and used to determine anomalies in prostate cancer on small tissue samples [42]. Protein biomarkers have also been useful for detecting metastasis because these biomarkers are accessible in the blood [43].

Computational biology has been a powerful tool in precision medicine and precision oncology, by which we can integrate various levels of data such as those produced from SNP screening and gene expression analyses [44,45]. Panomic analysis stems from genomics, transcriptomics, proteomics, and metabolomics and uses data to help further develop a treatment for diseases and create better means of diagnosis from the patient’s genetic variability, which are all essential to precision medicine [46]. Moreover, computational biology uses models to better understand the relationship between collected data [47,48]. SNP genotyping and gene expression profiling have circumvented the expenditures associated with analyzing the genome, transcriptome, and proteome of subjects. 

### 2.2. Breast Cancer

Breast cancer is the most common form of cancer among women in the world and is the second leading cause of cancer-related mortality after lung cancer [49]. A large number of risk factors for breast cancer, including various SNPs, thus has been identified, achieving the wide application of precision medicine (risk prediction) [23]. The most well-known mutations associated with breast cancer are those in the breast cancer susceptibility gene (*BRCA* 1 and 2), which result in the lifetime risk of developing breast cancer of up to 45–87% [10]. The Consortium of Investigators of Modifiers of *BRCA1* and *BRCA2* (CIMBA), established in 2006 [50], has provided a large number of *BRCA* mutations related to breast cancer. Women with these variants also have an increased risk of developing ovarian cancer [22]. Other representative genes related to breast cancer are summarized in Table 1.

Cancer genomes contain somatic mutations that develop over the lifetime of patients with cancer [51,52]. In oncogenesis, a group of these somatic mutations, “driver” mutations, allow cancer cells to gain a clonal selective advantage [51]. Driver mutations are one of the two biological classes of somatic mutations and are positively selected [53]. The other class of somatic mutations is called “passenger” mutations, which do not cause growth benefits to cancer cells but are inherited by cancer cells along with the cancer cell’s driver mutation. Somatic mutations in breast cancer and other types of cancers vary across tumor types and individuals [52]. Somatic driver mutations in breast cancer include mutations in the *TP53*, *PIK3CA*, *ATK1*, *CDH1*, *GATA3*, *PTEN*, *RB1*, *MLL3*, *MAP3K1*, *CDKN1B*, and *MAP2K7* genes [52,54,55]. The rates of these somatic driver mutations vary; some genes are more present in certain types of breast cancers than in others. Duplications in *ERBB2* (also called HER2) and deletions in *PTEN* or MAP2k4 were also noted in tumor genome. Additionally, novel mutated genes such as *TBX3*, *RUNX1*, *CBFB*, *AFF2*, *PIKER1*, *PTPN22*, *NF1*, *SF3B1*, and *CCND3* have also been identified [54]. The Cancer Genome Atlas Network reported that *PIK3CA* and *TP53* mutations were predominant in the mutation landscape of breast cancer cells. *PIK3CA* mutations were found in 40.1% of samples analyzed and *TP53* was found in 35.4% of samples [56]. Approximately 10% of the samples contained other mutations such as *MUC16*, *AHNAK2*, *SYNE1*, *KMT2C* (also known as *MLL3*), and *GATA3*.

The genome-wide association studies (GWAs) have also been contributing to the discovery of heritable cancer-causing variations [57]. As of 2020, >170 independent breast cancer susceptibility variants have been pinpointed because of GWAs. By identifying and continually discovering somatic mutations and variants in breast cancer, these findings can aid precision oncology in developing targeted therapies for patients with breast cancer. Using gene expression subtypes that can be established from these discoveries may help to advance the field of precision oncology.

Transcriptional variations are also able to identify breast cancer subtypes. In general, breast cancer tumors from the same subject have similar gene expression profiles compared to cancer tumors from other subjects [58]. This idea speaks to the heterogeneity of cancers and various expression profiles that can be found in different patients to explain their response to certain cancer drugs. Heterogeneity (spatial heterogeneity and temporal heterogeneity) exists in the same subject at the site of the cancer, affecting their response to cancer treatments [59]. By keeping track of cDNA microarrays, drug response can be predicted with further categorization and analysis. Tumors that are similar in their gene expression profiles can be grouped and tested to predict the outcomes for patients and individuals with similar tumor expression profiles.

Furthermore, common variants and gene expression profiles can be used to predict drug response in breast cancer [60,61]. These biomarkers are important in developing personalized diagnostics tools for predicting breast cancer risk and drug response. The U.S. Food and Drug Administration (FDA) approved Herceptin (trastuzumab) in 2006 for the treatment of approximately 30% of HER2-positive, node-positive breast cancer patients deemed unresponsive to standard medical protocols. They can also be used to further precision medicine initiatives in the field and create a library of common SNPs that can generate more accurate diagnoses and prognoses for breast cancer for patient groups.

**Table 1:** Representative genes responsible for breast cancer and found in glioma.

Type	Gene	Accession No.	Function
Breast	*BRCA1* *BRCA2*	NM_007294.4 NM_000059.4	Transcriptional regulator of DNA repair genes and tumor suppressor genes. *BRCA1* mutations are responsible for ~40% of inherited breast cancers and >80% of inherited breast and ovarian cancers. *BRCA1* and *BRCA2* variations can increase the lifetime risk of developing breast or ovarian cancer.
	*ATM*	NM_000051.4	This gene encodes a cell cycle checkpoint kinase that belongs to the PI3/PI4-kinase family. The normal function of this gene is to help repair DNA damage or kills the cell if it is unable to fix the damaged DNA.
	*TP53*	NM_000546.6	Halts the growth of cells with damaged DNA. *TP53* mutations are associated with various human cancers. The Li-Fraumeni syndrome, a complex hereditary cancer predisposition disorder, is mainly caused by germline mutations of this gene.
	*CHEK2*	NM_007194.4	The CHEK2 protein is a cell cycle checkpoint regulator and a possible tumor suppressor that is known to phosphorylate BRCA1. Mutations in this gene have been correlated with the development of Li-Fraumeni syndrome. This mutation increases the likelihood of predisposition to sarcomas, breast cancer, and brain tumors.
	*PTEN*	NM_001304717.5	Tumor suppressor gene that is mutated in a large quantity of cancers at high frequency. Helps regulate cell growth.
	*CDH1*	NM_001317185.2	Encodes epithelial cadherin or E-cadherin. When individuals inherit the mutated form of this gene, it causes *hereditary diffuse gastric cancer*, which can increase the risk of developing invasive lobular breast cancer in women. Mutations in this gene can also cause colorectal, thyroid, and ovarian cancers. Loss of function of *CDH1* increases tumor proliferation, invasion, and/or metastasis.
	*STK11* or *LKB1*	NM_000455.5	Encodes serine/threonine kinase 11 that regulates cell polarity and acts as a tumor suppressor. Mutations in *STK11* are associated with Peutz-Jeghers syndrome, which is characterized by the growth of polyps in the gastrointestinal tract, pigmented macules on the skin and mouth, and other neoplasms.
	*PALB2*	NM_024675.4	Encodes a protein that binds to *BRCA2*. *PALB2* may allow the stable intranuclear localization and accumulation of *BRCA2*.
	*BARD1*	NM_000465.4	Encodes protein that interacts with the N-terminal of BRCA1. Shares homology with the two most conserved regions of BRCA1, the N-terminal RING motif and the C-terminal BRCT domain. The RING motif is typically found in proteins that regulate cell growth. The protein encoded by *BARD1* may be the target of oncogenic mutations that are found in breast and ovarian cancer.
	*BRIP1*	NM_032043.3	The protein interacts with the BRCT repeats of *BRCA1*. The complex is important in the normal double-strand break repair activity of type 1 (*BRCA1*) breast cancers. *BRIP1* may be a target of germline cancer-inducing mutations.
	*CASP8*	NM_001372051.1	Encodes a member of the cysteine-aspartic acid protease (caspase) family. This protein allows for the apoptosis induced by Fas. Associated with the risk of developing cancer [62].
	*CTLA4*	NM_005214.5	A member of the immunoglobin gene superfamily. Encodes a protein that sends an inhibitory signal to T cells. Expressed in some cancer cells [63].
	*FGFR2*	NM_000141.5	The protein encoded by this gene is a member of the fibroblast growth factor receptor family, where amino acid sequence is highly conserved. Aberrations in *FGFR2* have been seen to affect *FGRFR2* signaling that has been recognized in breast cancer. Amplification of *FGFR2* is present in 3.6% of triple-negative breast cancers (TNBCs) [64].
	*H19*	NR_002196.2	Gene only expressed from maternally inherited chromosome. Encodes a non-coding RNA that functions as a tumor suppressor. Mutations in *H19* are associated with the development of Beckwith-Wiedemann Syndrome and Wilms tumorigenesis.
	*MRE11A*	NM_05591.4	Encodes a nuclear protein involved in homologous recombination, telomere length maintenance, and DNA double-strand break repair. This protein is a member of the MRE11/RAD50 double-strand break repair complex composed of 5 proteins.
	*NBN*	NM_002485.5	Mutations in *NBN* are associated with the development of Nijemegen breakage syndrome that is characterized by cancer predisposition, microcephaly, growth retardation, and immunodeficiency. The gene product of *NBN* has been proposed to be involved in DNA double-strand break repair and DNA damage-induced checkpoint activation.
	*RAD51*	NM_002875.5	Encodes a protein important for repairing damaged DNA. The protein is a member of the RAD51 family. It interacts with single-strand DNA-binding protein RPA and RAD52. This protein is also thought to be involved in homologous pairing and strand transfer of DNA. It interacts with *BRCA1* and BRCA2. BRCA2 inactivation can result in the loss of RAD51 controls and be an important event resulting in genomic instability and tumorigenesis.
	*TERT*	NM_198253.3	Encodes one subunit of the enzyme telomerase that lengthens telomeres at the end of chromosomes. The lengthening of the cancer cell telomeres allows them to continually survive.
	*TOX3*	NM_001080430.4	This gene encodes a protein that holds an HMG-box. This protein is possibly engaged in bending and unwinding DNA and modulating chromatin structure because of the HMG-box. This gene’s minor allele has been associated with a higher risk of developing breast cancer.
Glioma	*AVIL*	NM_006576	Encodes a member of gelsolin/villin family of actin regulatory proteins. May contribute to the development of ganglia. *AVIL* expression is increased in glioblastomas as well as glioblastoma stem/initiating cells [65]. Patients with an increased level of *AVIL* expression seemed to have a worse prognosis [65].
	*MMP9*	NM_004994.3	The matrix metalloproteinase (MMP) breaks down the extracellular matrix. *MMP9* is a member of the MMP family involved in disease processes like metastasis and possibly in tumor-associated tissue remodeling.
	*FN1*	NM_212482.4	Encodes fibronectin, a glycoprotein present in a soluble dimeric form in plasma, and in a dimeric or multimeric form at the cell surface and in extracellular matrix. Fibronectin is known to be involved in cell adhesion and migration progresses such as metastasis.
	*COL3A1*	NM_000090.4	Encodes the pro-alpha1 chains of type III collagen found in skin, lungs, intestinal walls, and the walls of blood vessels. Mutations in this gene are associated with the development of Ehlers-Danlos syndrome type IV.

### 2.3. Glioma

Gliomas are a type of tumor originating from the glial cells and can be categorized into four groups by the World Health Organization (WHO) 2007 classification: low-grade, consisting of grades I and II, and high-grade, consisting of grades III and IV [66]. The severity and the dismal prognosis of these tumors increase as the grades move from I to IV, although there may be a need for updating this classification [67]. Grade I gliomas usually develop in children, some grade II gliomas invade healthy tissue in the brain slowly, grade III gliomas are more malignant and can spread to healthy tissue in the brain, and grade IV gliomas are the most aggressive and survive and flourish through angiogenesis. Glioblastoma multiforme (GBM) is an aggressive and rare form of grade IV primary brain tumors that usually have a dismal prognosis, and the search for effective GBM treatments is underway [68]. The glioma formation can sometimes be attributed to hereditary diseases like Turcot syndrome, Li-Fraumeni syndrome, or neurofibromatosis [69], but other heritable variations (SNPs) have been identified in various populations (Table 1). In the Iraq population, SNPs in interleukin (IL)-10, -12p40, and -13 genes have been identified as predictors of susceptibility to glioma [70]. Polymorphisms in AKAP6 have been acknowledged to increase the risk of developing glioma in patients, including high-grade glioma in Han Chinese adults [25]. In Brazil, patients with the “CAGT” haplotype of KDR SNPs were observed to have an increased risk of developing grade IV glioma with its properties in spurring angiogenesis [26]. Again, haplotyping is important in determining the risk of glioma, but there also have been studies that identified specific SNPs that reflect the aggressiveness of gliomas as well.

Gene expression profiles can help classify various types of brain tumors, including gliomas, and further identify potential therapeutic targets [12]. *AVIL* expression is increased in glioblastoma cells and glioblastoma stem or initiating cells [65]. This gene contributes to the prognosis of patients with glioblastoma as its overexpression leads to cell proliferation and migration. Patients who have a higher expression of *AVIL* tend to have a worse prognosis. When *AVIL* is silenced in culture, glioblastoma cells are almost eliminated, and silencing of *AVIL* in in vivo xenografts in mice inhibits these glioblastoma cells [65]. The study showed the possibility of FOXM1 regulation of LIN28B mediating the tumorigenic effect of *AVIL*. Integrin pathways in gliomas are inferred to be responsible for their characteristic behaviors such as migration and invasion [12]. Specific genes shown in the risk of developing gliomas are the *MYC* oncogene and others [12]. In this way, precision measures of diagnostics and treatment can be possible. Scientists have identified 34 genes expressed in GBM in vitro [71]. These genes are responsible for the diffusion and infiltrating characteristics of GBM that makes it a dismal form of brain cancer [71]. Although further studies are needed to expand these findings to clinical applications, these genes are interesting targets for developing novel therapies.

In patients with glioma, tumor-infiltrating immune cells (TIICs) that transform low-grade glioma to high-grade glioma are relevant to the clinical outcome of glioma [72]. The TIICs may be used as a biomarker to predict the effect of drug treatment and survival of certain patients under chemotherapy and immunotherapy [73]. Additionally, multiple TIICs and bulk tumor transcriptome data were used to predict the clinical outcome of patients with colon cancer [74]. The TIIC biomarkers can be a promising supplementary avenue in precision oncology for patients with gliomas. Similarly, adult high-grade gliomas (HGGs) and pediatric HGGs appear identical phenotypically, but when scientists investigated SNPs using next-generation sequencing (NGS), some important genetic differences were identified, which in turn impacts the type of treatments [48]. In 2011, a major discovery was made within another chemotherapeutic research initiative: vemurafenib, a fragment-derived BRAF protein kinase inhibitor, was approved as a cancer treatment for BRAF-mutant melanoma. Since then, continued enthusiasm within the precision medicine field has intensified, and research into directed medicinal approaches continues. Bevacizumab (Avastin), a vascular endothelial growth factor (VEGF) inhibitor, has been used as a targeted drug therapy to treat glioblastoma, but the clinical response to this drug is highly variable [75]. This drug is administered intravenously and stops new blood vessels that supply blood to the tumors from forming. The deprivation of blood to these tumors results in the death of tumor cells.

## 3. Machine Learning—A Keystone That Paves the Way for Precision Oncology

### 3.1. Overview

AI is a promising tool in the development of precision medicine and precision oncology, although it has not yet contributed to the clinical outcomes. At the preclinical level, ML, a subfield of AI in which computers learn through experience, has been used as a powerful tool to predict cancers using pattern recognition and to track cancers over a lengthy period. Like precision medicine, the concept of ML has a long history—Alan Turning, a British mathematician, predicted the reality of ML in the 1950s. Subsequently, in 1952, Arthur Samuel, the father of ML, developed the first ML programs that played checkers. This computer improved through its experiences of playing checkers through a series of trial-and-error efforts and used mistakes to continuously improve its performance. Since then, ML algorithms have continued to evolve, and their relevant applications have increased over the last 70 years.

The ML algorithms are either supervised, semisupervised, or unsupervised. Supervised ML requires labeled training data for the training, whereas unsupervised ML can be trained by unlabeled data since this ML approach identifies the hidden structure of data using algorithms such as clustering [76]. Semisupervised ML uses both approaches, which enables us to reduce the high cost of labeling data. In addition to this general classification, many different ML algorithms can be employed in precision medicine and precision oncology (Table 2). Among them, the artificial neural networks (ANNs) have made an early success in this field. The basis of ANNs was formed from a model of neuronal interaction outlined in the book *The Organization of Behavior* by Donald Hebb in 1949. Hebbian learning, the concept of strengthening neuronal connections through synchronous activation and the weakening caused by diachronous neuron activation, has been the basis of the ANN. Nodes in the ML neural network represent neurons in the human brain, and the resulting strength or weakness of those nodes are defined as “weights”, which can be either “positive” or “negative”.

ML has become increasingly important in the field of precision medicine and precision oncology since it can identify patterns in biomarkers across various datasets to find the specific pattern associated with the risk of cancer or the effect of drugs [77,78]. An important application of ML in precision oncology is the image-based digital diagnosis to classify various types of cancers performed by microscopic tissue pathological analysis [79]. Magnetic resonance imaging (MRI) data can also be used to classify a variety of cancers combined with other genetic tests [80]. Risk prediction for developing certain cancers, based on identified biomarkers and “molecular signatures”, can also be determined using ML.

Importantly, ML not only is useful for analyzing a single biomarker but also is enabling the integration of various datasets such as images and genomic information. The delta-radiomics is proposed as a biomarker to help in predicting cancer treatment outcomes [81]. Some recent studies reported that ML predicts somatic mutation and chromosomal instability of tumors from images [82,83]. Thus, ML is expected to become more and more important as the development of multimodal characterization of tumor cells continues.

**Table 2:** Representative machine learning algorithms used in precision oncology.

Algorithm	Characteristics
K-nearest neighbor (KNN)	Often described as the simplest ML algorithm; no training phase is required.
Support vector machine (SVM)	Simple structure and high generalization capability; works well with insufficient training data [84].
Artificial neural network (ANN)	Mimics neuronal network, in which each node changes the connection strength by experience.
Decision tree (DT) learning	A popular tree-based method for classification and regression, in which the learned model is represented as a decision tree.
Naive Bayes (NB)	Probabilistic classifier that treats each feature variable as an independent variable.
Bayesian network (BN)	A probabilistic graphical model in which a directed acyclic graph represents potential causal relationship between variables.

### 3.2. Bayesian Networks

**Figure 2:**
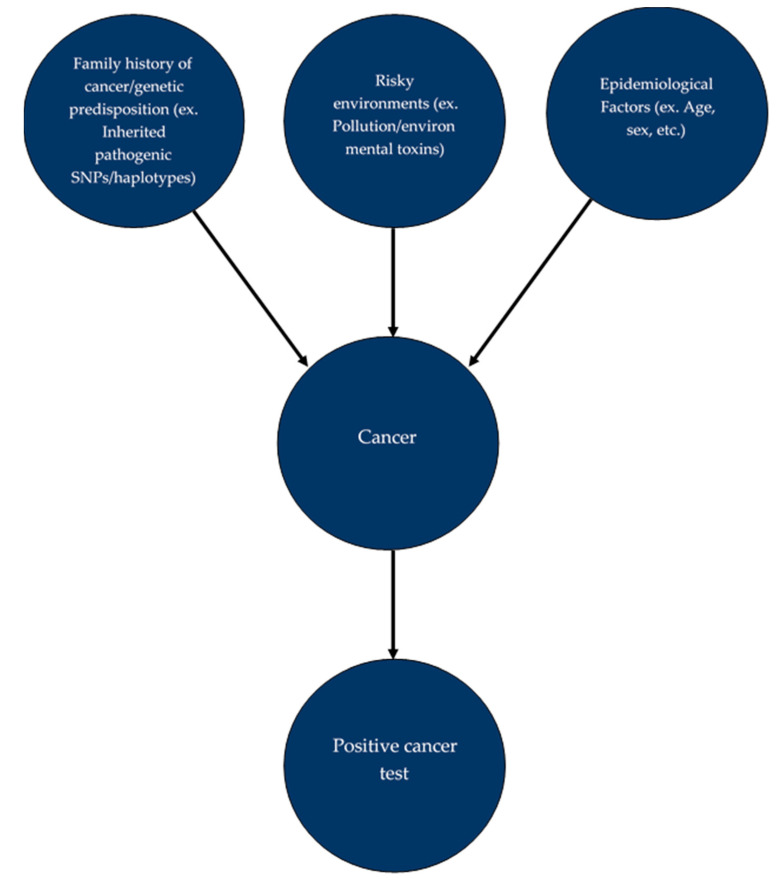
Simple pictorial of a Bayesian network without probabilities.

Bayesian networks (BNs) are ML algorithms that have a high potential to be the leading strategy in precision medicine and precision oncology (Figure 2). At its core, the BN is subjective and can be varied easily to meet the expectations of its creator compared to the more widely used regression-based models; BNs thus have the flexibility and the ability to work with incomplete information in times of uncertainty, allowing physicians to make decisions using various patients’ data including SNPs and other genetic, environmental, and epidemiologic components [78,85,86]. BNs can also be used to determine probability calculations with Bayesian inference and are thus useful to find potentially responsible factors from SNPs and other variables such as environmental risks and epidemiological factors. Furthermore, BNs can be used not only for predicting the risk of developing cancer for the first time but also to predict the risk of cancer recurrence [87], although other regression-based methods described in this review, in principle, can be used for this purpose. Since BNs are still relatively new to the medical field compared to the regression-based models, there have been comprehensive tutorials on how to use BNs in the medical field [88].

BNs have been used in predicting hematological malignancies, acute myeloid leukemia (AML), and myelodysplastic syndrome (MS), using expression profiles [86], achieving a high level of accuracy (93%) and precision (98%) compared to other methods. Combining BNs with other statistical methods, including graphical lasso, has produced more accurate prognosis predictions for certain cancers [89]. Gene expression profiles can be used in BN to perform specific risk predictions for cancers as “molecular signatures”. In the case of triple-negative and medullary breast, ovarian, and lung cancers, key genes, including proline-rich protein 1A, were modeled using a BN [89]. Another example is its use for predicting pancreatic cancer early and determining how cancer will respond to certain treatments [81]. While there are still limitations in using delta-radiomics in precision oncology, delta-radiomic features that do not necessarily rely on these image sets can still provide a substantial form of analysis [81].

Although using BNs has produced positive results in cancer and disease prediction, this algorithm is not always the best tool. In using BN to predict breast cancer recurrence in women in the Netherlands Cancer Registry compared to other statistical methods, including logistic regression, for risk prediction, researchers found that logistic regression was just as accurate if not more accurate at predicting compared to their developed BN [90].

### 3.3. ML in the Treatment of Breast Cancer and Glioma

The accumulated data have facilitated the application of ML to the studies of breast cancer and glioma. Histological images along with the mammogram and MRI data have been frequently used in the ML-based analysis in precision oncology compared to the genomic data. The Wisconsin Breast Cancer dataset is a commonly used dataset containing 569 instances of breast cancer. ML studies using this, and many other image datasets have identified the most accurate ML approaches for classifying cancer [91,92]. A meta-analysis including 11 articles found that the SVM is the best algorithm for accurately predicting the risk of breast cancer from images among compared algorithms, including ANN, decision tree (DT), NB, and K-nearest neighbors (KNN) [93]. Two ML models, the Breast Cancer Risk Assessment Tool (BCRAT) and Breast and Ovarian Analysis of Disease Incidence and Carrier Estimation Algorithm (BOADICEA), have already been integrated into clinical guidelines [94,95].

Bayes theorem has also been applied to breast cancer to evaluate the prognosis [96]. In this study, three methods were employed. The two methods that had the greatest results were the best decision integration model and the best partial integration model [96]. In decision integration, separate models are created for the two datasets (clinical and microarray data), which are later combined to predict the outcome probabilities. The best partial integration incorporates both the structures for clinical data and microarray data combined with the common ground of having the same outcome. These methods of employing BNs show a promising approach for using BNs to determine the likelihood of outcomes for patients not only with breast cancer but also with other forms of cancer by inputting data that are specific to those cancers [96].

Glioma is also a target of the novel ML approach, in which ML algorithms are used to predict the risk of developing gliomas of various grades. The complement NB classifier was used in this study to determine the different genes observed in various stages of gliomas [97]. Some ML algorithms such as the random forest and complement naive Bayes showed up to 97.1% accuracy for predicting grade I to II gliomas and an 83.2% accuracy for grade II to IV gliomas using gene expression patterns. CNB presented an accuracy of 72.8% for grade II to III gliomas. Another study identified 11 genes that may be useful for classifying glioblastoma [98]. ML algorithms can increase accuracy in diagnosis and further develop targeted therapies for treating gliomas, especially higher-grade gliomas that, at times, are hard to treat. ML is suitable as a personalized diagnostic tool for cancer risk prediction [99].

## 4. Future Directions

ML will continue to be the most powerful method in precision medicine and precision oncology with promising improvements in the accuracy of predicting risks and treatment outcomes. An expected future direction is the one toward treatment—from diagnosis to pharmacogenetics and pharmacogenomics. The distinction between pharmacogenetics and pharmacogenomics lies within the scope of genetic analyses under assessment. Pharmacogenetics is defined as the study of variability in drug response due to heredity, largely related to specific genes impacting drug metabolism, while pharmacogenomics is a considerably broader term that encompasses the entirety of the genome and its potential holistic impact on drug response [100].

This movement would further accelerate the utilization of various levels of information in ML. Although the holistic understanding of the human genome will indeed bolster understandings of variations within drug responses, many ML-based approaches in precision oncology have used histological images of tumors, which provide limited information. Effective utilization of information about genetic mutations and/or histone modifications will lead to better treatment outcomes. Additionally, factors such as patient’s age, underlying diseases of organs, pregnancy, and mutations, can cause some pharmacokinetic variations and impact drug absorption, distribution, metabolism, and excretion. Precision oncology using ML takes all factors into account when performing precision diagnosis and creating chemotherapy that works best for patients. Although precision medicine is still a work in progress, it can someday be beneficial and address many issues, including adverse side effects or insufficient drug effectiveness, that are sometimes observed in a one-size-fits-all model for drugs.

One main challenge with creating a personalized approach to oncology, risk prediction, and monitoring the drug response of patients is the security of the data and the privacy of patients [21]. With the vast amount of information and -omics datasets that are stored for quantitative analysis, this event can increase the risk of data leakage, which can be a problem, especially when there are patient identifiers attached to the data. This event can be a violation of the Health Insurance Portability and Accountability Act (HIPAA).

In some trials, there has also been little success in treating patients with targeted therapeutics [101]. Although there is abundant research supporting the success of precision medicine and precision oncology, specifically, there still needs to be more research into its efficacy. Booth et al. found that ML-based determination of glioma imaging biomarkers (with MRI data) could accurately classify brain tumors using SVM recursive for classification, but ML has yet to prove itself compared to other standard statistical methods [102]. Their finding was that for ML to work effectively and to have the most advantage, extensive, well-annotated datasets across multiple centers need to be used [102]. This concept reinforces the idea of the importance of having ample data available for computers to produce the best results and predictions with high accuracy. New methods have been proposed to combat some of these limitations. With more data available, we can build better systems that can diagnose and treat patients in a more specific way, using quantitative and statistical methods and ML to detect patterns.

## 5. Conclusions

Various quantitative and qualitative techniques can be used to assess the risk of patients developing different types of cancers. Some of these techniques, including computational biology, ML, and BNs, have achieved significant success in precision medicine and precision oncology, especially in breast cancer and glioma. These methods can be used to propel the field even further and determine the prognosis of cancer.

Beyond the genomic data, many factors account for pharmacokinetics and pharmacodynamics in patients. Although tumor images, as well as SNPs and gene expression profiles to a certain extent, have propelled the research of precision oncology, precision oncology still needs to incorporate more information to provide patients with optimal therapies with minimal side effects. Early risk prediction using genetic data as well as the incorporation of epigenetic and environmental information using ML will make better predictions and find effective treatments for patients.

## Data Availability

No new data were created or analyzed in this study. Data sharing is not applicable to this article.
